# Label-free detection of cellular drug responses by high-throughput bright-field imaging and machine learning

**DOI:** 10.1038/s41598-017-12378-4

**Published:** 2017-09-29

**Authors:** Hirofumi Kobayashi, Cheng Lei, Yi Wu, Ailin Mao, Yiyue Jiang, Baoshan Guo, Yasuyuki Ozeki, Keisuke Goda

**Affiliations:** 1 Department of Chemistry, University of Tokyo, Tokyo, 113-0033 Japan; 2 Department of Computational Biology, Carnegie Mellon University, Pittsburgh, Pennsylvania 15213 USA; 3 Department of Electrical Engineering and Information Systems, University of Tokyo, Tokyo, 113-8656 Japan; 4 Japan Science and Technology Agency, Kawaguchi, 332-0012 Japan; 5 Department of Electrical Engineering, University of California, Los Angeles, California 90095 USA

## Abstract

In the last decade, high-content screening based on multivariate single-cell imaging has been proven effective in drug discovery to evaluate drug-induced phenotypic variations. Unfortunately, this method inherently requires fluorescent labeling which has several drawbacks. Here we present a label-free method for evaluating cellular drug responses only by high-throughput bright-field imaging with the aid of machine learning algorithms. Specifically, we performed high-throughput bright-field imaging of numerous drug-treated and -untreated cells (N = ~240,000) by optofluidic time-stretch microscopy with high throughput up to 10,000 cells/s and applied machine learning to the cell images to identify their morphological variations which are too subtle for human eyes to detect. Consequently, we achieved a high accuracy of 92% in distinguishing drug-treated and -untreated cells without the need for labeling. Furthermore, we also demonstrated that dose-dependent, drug-induced morphological change from different experiments can be inferred from the classification accuracy of a single classification model. Our work lays the groundwork for label-free drug screening in pharmaceutical science and industry.

## Introduction

In the last decade, high-content screening based on multivariate single-cell imaging has been proven effective in drug discovery to evaluate drug-induced phenotypic variations in gene expression, protein localization, and cytoskeletal structure^1,2^. A number of studies have shown that cellular responses to drugs or even the mechanism of action for unknown compounds can be correctly predicted with such variations^3–5^. The primary advantage of image-based screening over conventional univariate screening is its capability of multivariate profiling with a large number of variables by which leading compounds can be identified with high sensitivity^6^. Moreover, with advances in molecular biology, image-based screening has been coupled with RNA interference or gene-modified cell lines to provide further information on cellular responses to drugs^7^. In a recent study, for instance, a library of fluorescently tagged reporter cell lines has been produced to find an optimal cell line for image-based screening^8^.

Unfortunately, conventional multivariate single-cell imaging for high-content screening falls short in addressing the full needs of the drug discovery community as it inherently requires fluorescent labeling which has several drawbacks. First of all, fluorescent probes are not available for all target molecules and may interfere with natural cellular functions^9^. While a wide range of immunofluorescent probes are commonly used in single-cell imaging for multivariate profiling, they are costly and require time-consuming labeling processes, including cell fixation which kills the cells, hindering large-scale assays^10^. Fluorescently tagged cell lines can offer live-cell assays without the labeling process, but the development of such cell lines requires more effort than immunofluorescent labeling^8^. Therefore, an alternative for image-based high-content screening without the need for fluorophores is clearly needed for easy manipulation and economical assays.

In this paper, to avoid the above limitations, we present a method for evaluating cellular drug responses only by high-throughput bright-field imaging with the aid of machine learning. This was made possible by acquiring a large number of bright-field images of numerous drug-treated and -untreated cells (N = ~240,000) by optofluidic time-stretch microscopy with high throughput up to 10,000 cells/s and using the label-free cell images and machine learning to identify their morphological variations which are too subtle for human eyes to detect. Consequently, we successfully identified drug-treated and -untreated cells with a high accuracy of 92% without the need for any labeling techniques. Specifically, we used MCF-7 as a model cell line and paclitaxel, an anti-cancer drug, as a model drug to induce morphological change to the cells. We quantitatively analyzed the morphological change of paclitaxel-treated MCF-7 cells compared with a negative control, or untreated cells. The degree of the morphological change, inferred from classification accuracy, increased with the drug concentration and treatment time, suggesting that the morphological change observed from bright-field images can be utilized as an indicator for drug discovery. Our work lays the groundwork for label-free drug screening in pharmaceutical science and industry.

## Results

### Workflow for detection

**Figure 1:**
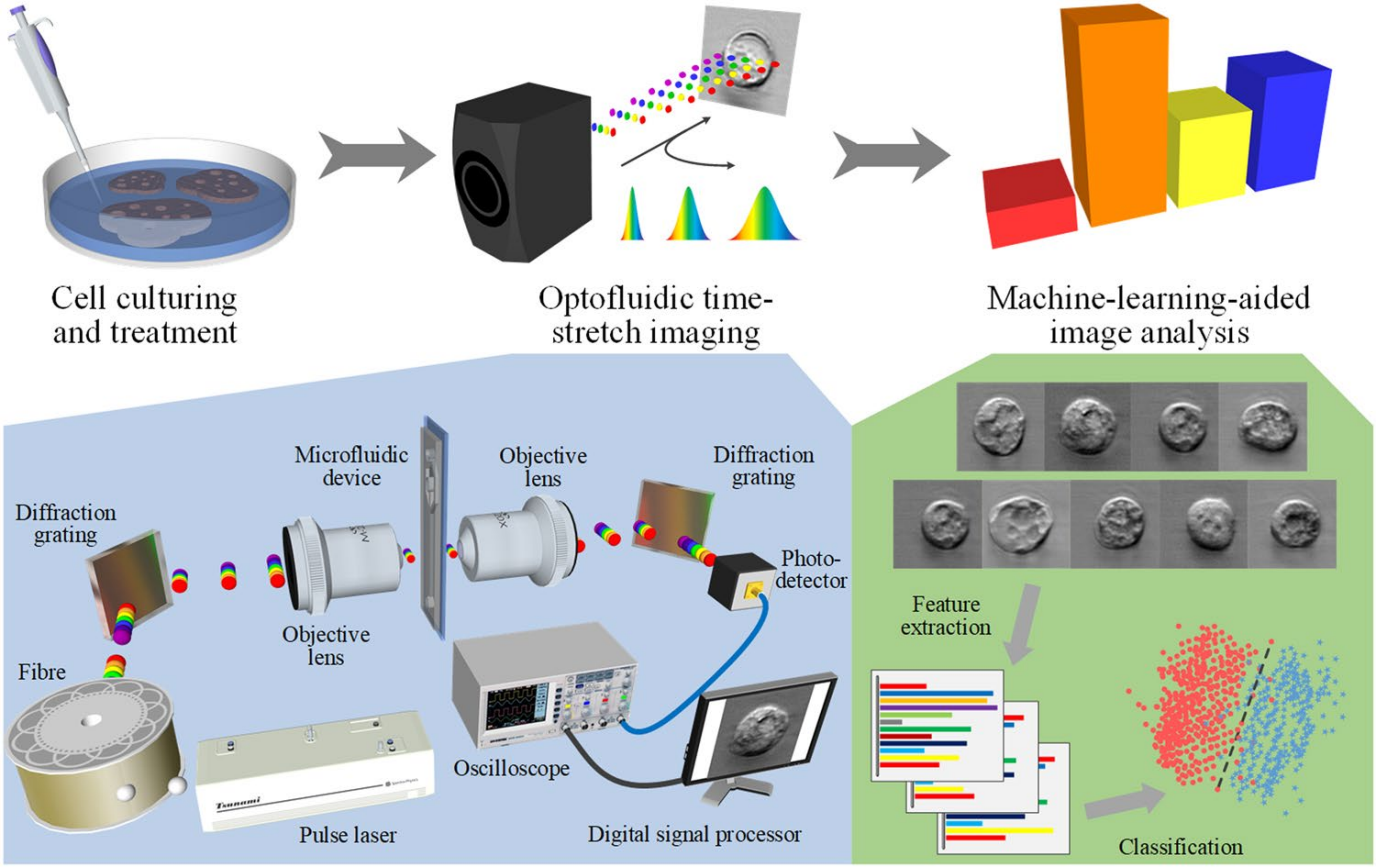
Workflow of the label-free detection of drug-induced morphological variations in cancer cells with optofluidic time-stretch microscopy. The insets show the schematics of optofluidic time-stretch microscopy and machine-learning-aided image analysis. As schematically shown in the figure, the procedure of our method can be divided into three parts: (i) cell culturing and treatment, (ii) optofluidic time-stretch imaging, and (iii) machine-learning-aided image analysis. In the first part, cells of interest are cultured and treated by a drug. In the second part, the treated cells are subject to high-throughput bright-field imaging. In the last part, machine learning algorithm is applied to the images for the identification of their morphological variations induced by drug treatment. While the morphological change in a single cell is miniscule, the large number of bright-field single-cell images can render the morphological change discovered by machine learning statistically significant and robust.

As schematically shown in Fig. 1, the procedure of our method can be divided into three parts: (i) cell culturing and treatment, (ii) optofluidic time-stretch imaging, and (iii) machine-learning-aided image analysis. In the first part, cells of interest are cultured and treated by a drug. In the second part, the treated cells are subject to high-throughput bright-field imaging. In the last part, machine learning algorithm is applied to the images for the identification of their morphological variations induced by drug treatment. While the morphological change in a single cell is miniscule, the large number of bright-field single-cell images can render the morphological change discovered by machine learning statistically significant and robust.

### Optofluidic time-stretch microscope

**Figure 2:**
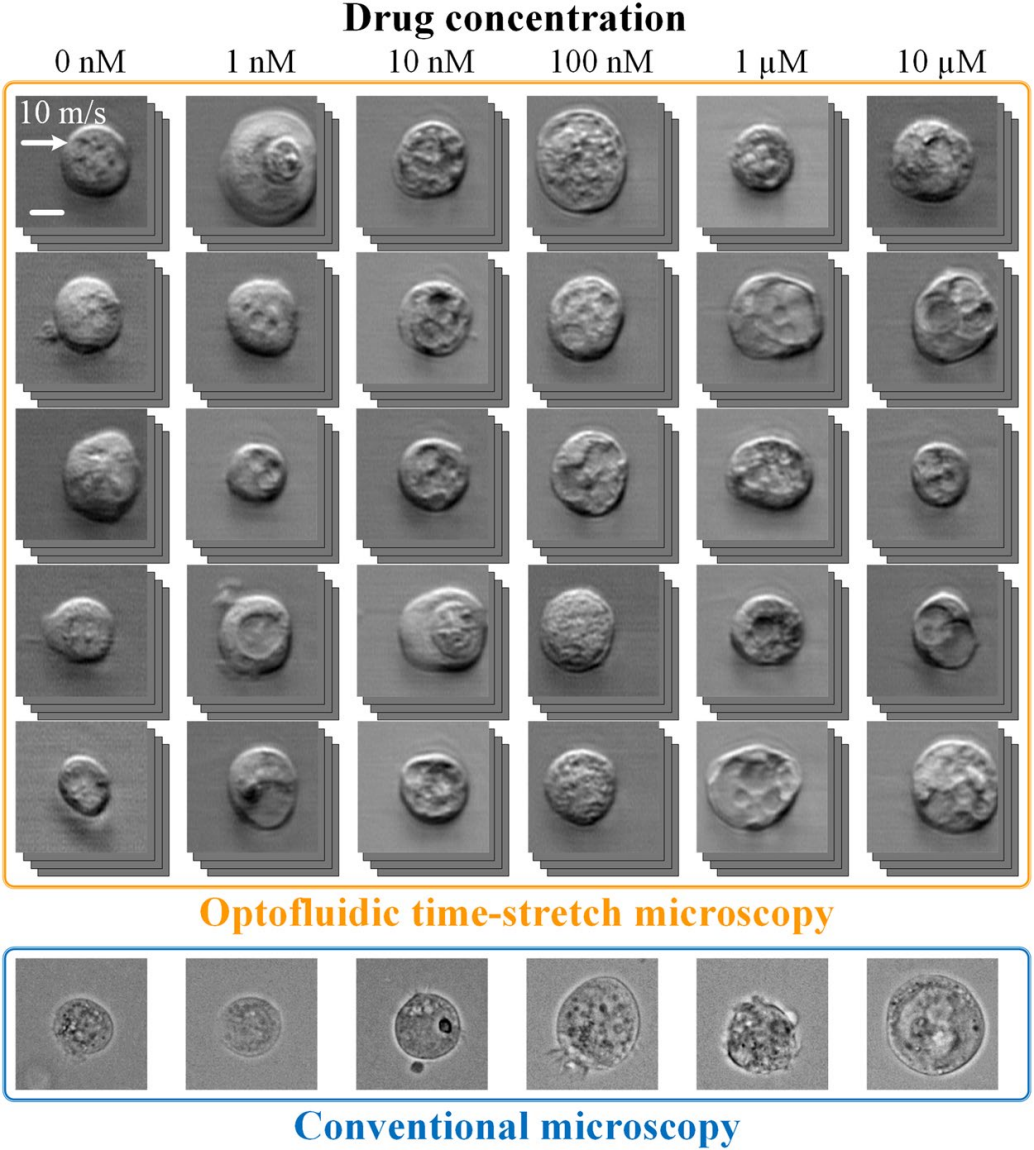
Image libraries of drug-treated and -untreated MCF-7 cells under our optofluidic time-stretch microscope (flowing at a speed of 10 m/s). Compared with the static images obtained by a conventional microscope, despite the high flow speed, the optofluidic time-stretch microscope can acquire blur-free images of cells with decent image quality. Scale bars: 10 µm.

To highlight the advantages of our optofluidic time-stretch microscopy in acquiring high-quality bright-field images of cells in a high-speed flow, we show two image libraries including drug-treated and -untreated MCF-7 cells under a conventional microscope and our optofluidic time-stretch microscope in Fig. 2a and b, respectively. The optofluidic time-stretch images clearly show the fine structures in the cells with sufficient contrast, at an equivalent level of which a conventional light microscope can achieve. In addition, both static and optofluidic time-stretch images demonstrate consistent cellular morphology. Note that each blur-free optofluidic time-stretch image was obtained in 4 µs (corresponding to the frame rate of 250,000 frames/s), while maintaining the same pixel resolution as those taken by a CMOS camera on a conventional microscope. The high pixel resolution of our optofluidic time-stretch microscope retains the rich cellular information in the acquired images. Furthermore, the high frame rate of our system enables high-throughput imaging, which favours the application of machine learning techniques for mining the cellular information in the images as these techniques typically require a large volume of training data for obtaining an accurate prediction model. With the advantages of our optofluidic time-stretch microscope, we overcame the challenges of evaluating cellular drug responses only by bright-field imaging.

### Classification of drug-treated and -untreated cells

**Figure 3:**
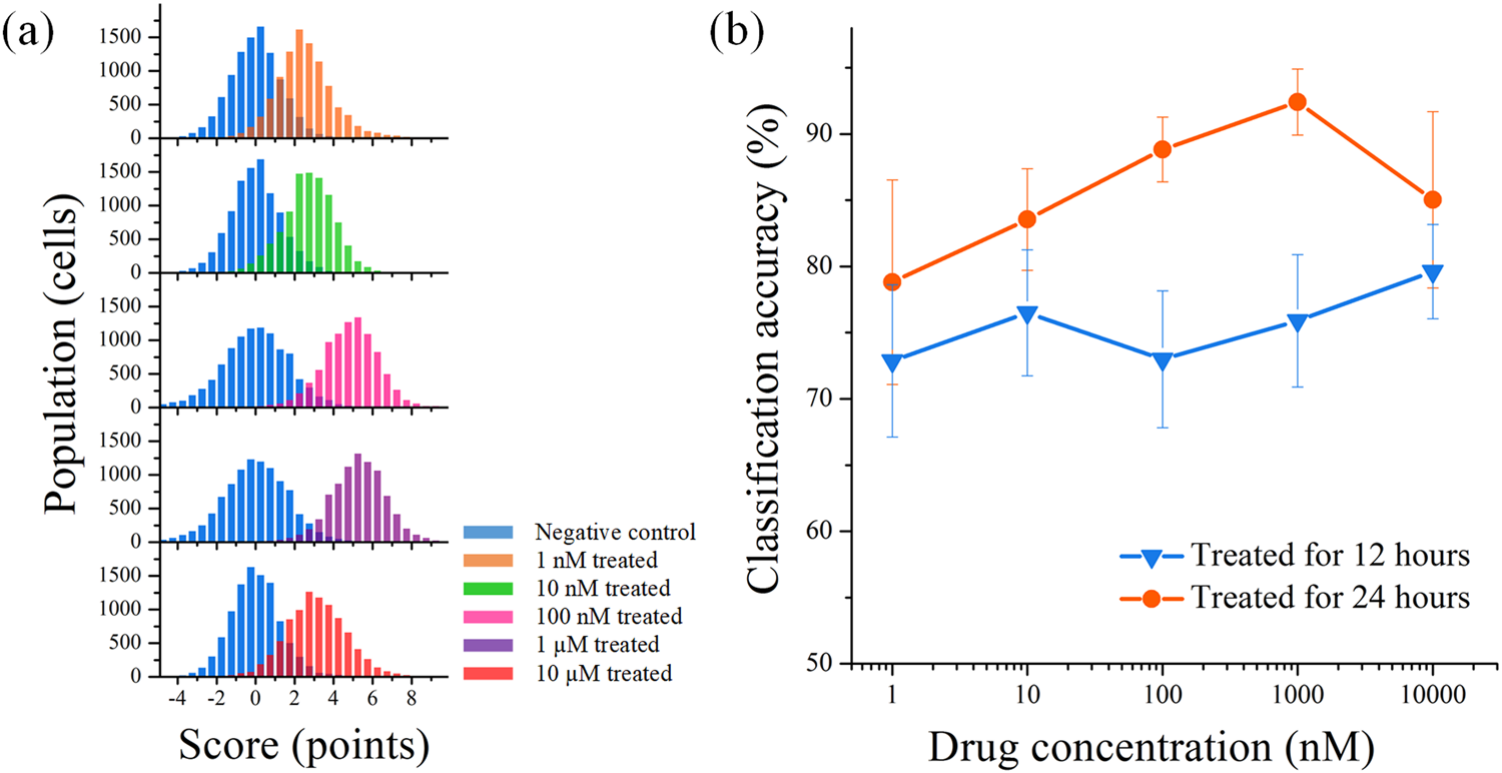
Classification of drug-treated and -untreated cancer cells. (**a**) Histograms of SVM classification scores for MCF-7 cells treated with various concentrations of paclitaxel for 24 hours. Each population consists of up to 10,000 cells. (**b**) Classification accuracy at various drug concentrations and incubation times. The error bars represent standard errors of the cross-validation estimation of average classification accuracy (n = 4).

To quantitatively evaluate the impact of an anti-cancer drug concentration on the morphological change of cancer cells, we treated MCF-7 cells with various concentrations of paclitaxel for 24 hours. We used a support vector machine (SVM)^11^ aimed to find a hyperplane that separates with a large margin between two classes of data and to classify the negative control and each drug-treated population. Figure 3a shows the distribution of classification scores between the two populations. As we use a linear kernel for the SVM classification, the classification score is given by

1 $$Y={\boldsymbol{w}}\cdot {\boldsymbol{x}}+b,$$

where ***w*** is the normal vector to the hyperplane, representing the weight assigned to each feature, ***x*** is the test data, and *b* is the bias. The values of ***w*** and *b* are provided in the Supplementary Data. The separation between the two populations becomes larger as the drug concentration increases up to 1 µM, indicating that features corresponding to each class (drug-treated or negative control) become more distinct. Given that extracted features correspond to morphological change, the SVM classification suggests a dose-dependent drug-induced morphological change. This dose-dependent change was further supported by four trials of SVM classification as shown in Fig. 3b, where higher classification accuracy equates to larger separation between the two populations. The classification accuracy here is defined by *A* = (*X*_1_ + *X*_2_)/*N*, where *X*_1_ and *X*_2_ are the numbers of correctly assigned incidences and *N* is the total number of test data points. The accuracy range is from 50% (random) to 100% (perfect). We note that classification accuracy reaches its maximum at 1 µM for 24-hour drug-treated cells and then drops at 10 µM. The drop at 10 µM is possibly due to the presence of the high concentration of DMSO (1% v/v). Next, we evaluated the impact of drug-treatment time on morphological change. We performed the same dose-ranging experiment with a 12-hour drug treatment whose classification accuracy evolution is shown in Fig. 3b. The classification accuracy curve shows a relatively monotonic increase, but lower accuracy at each drug concentration than that of the cells treated for 24 hours. This result illustrates that shorter treatment time induces less discrepancy in the features of each class, hence making it more difficult to identify drug-induced morphological change. Accordingly, we use the data acquired from the 24-hour treatment on the quantitative analysis shown below.

### Dose-dependent change in feature space

**Figure 4:**
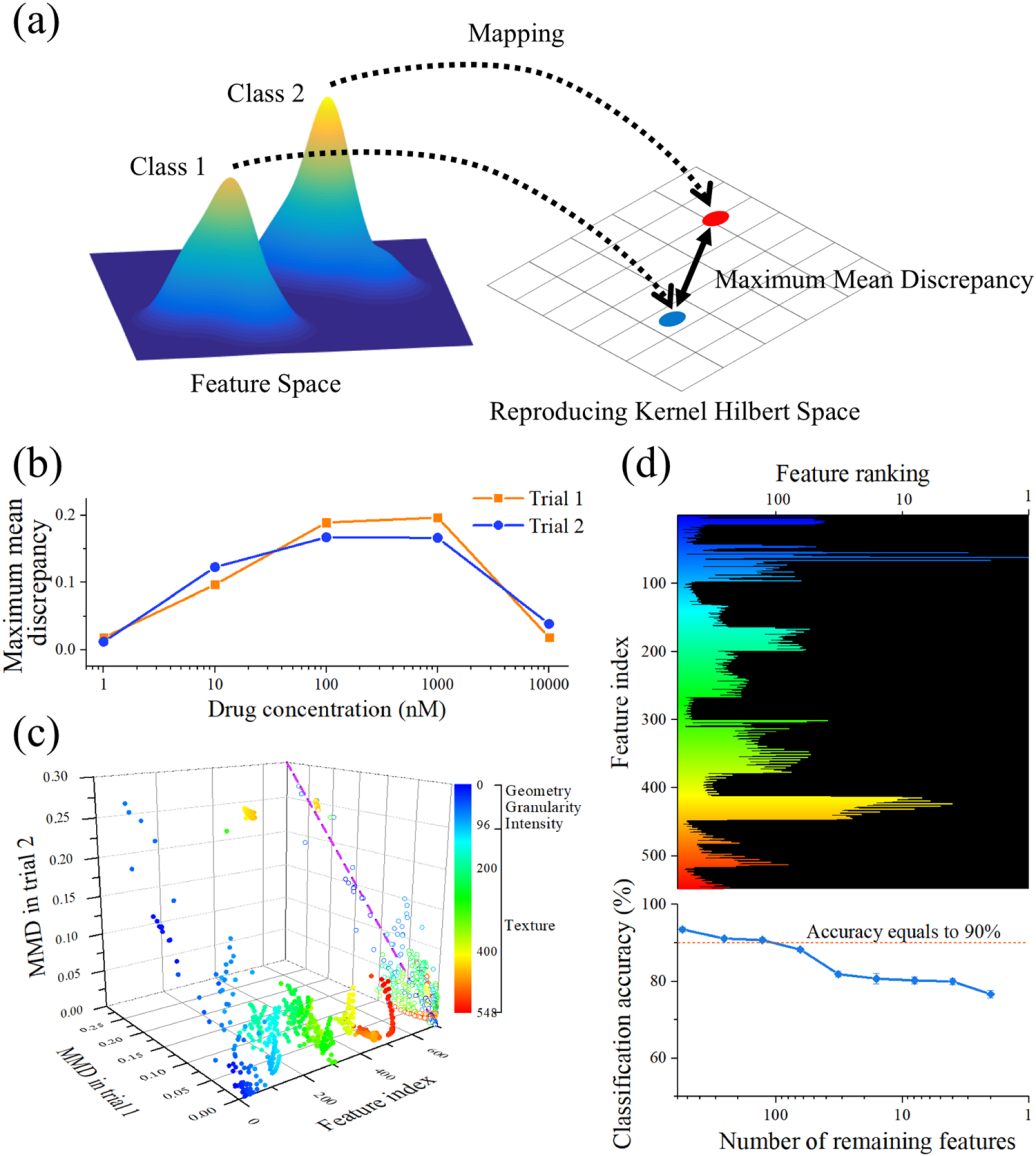
Calculating maximum mean discrepancy (MMD) between the negative control and drug-treated cell population. (**a**) Illustration of the maximum mean discrepancy (MMD). (**b**) MMD between the negative control and drug-treated cell population at each drug concentration. Trial 1: data from the first experiment. Trial 2: data from the second experiment. (**c**) MMD of each feature in trial 1 at 1 µM and trial 2 at 100 nM. At these concentrations, the MMD in the whole feature space is the largest in each experiment. Features with a higher score of the MMD in both trials are highly correlated, indicating that the significant features were consistent in both experiments. The color scale represents feature index, showing types of morphological changes that undergo larger scores of the MMD. (**d**) Classification accuracy with a reduced number of features. Lower MMD features were removed based on the ranking of the MMD for each feature in the classification between the negative control and the dataset at 1 µM in the first experiment (top). The classification accuracy was maintained over 90% with more than 100 features (bottom). The color scale is consistent with that in Fig. 4c. Feature ranking and the number of remaining features are illustrated in logarithmic scale. The error bars represent standard errors of the cross-validation estimation of average classification accuracy (n = 10).

In order to analyze the amount and type of morphological changes in the feature space, we calculated the maximum mean discrepancy (MMD)^12^ between the populations of drug-treated and -untreated cells at each drug concentration. Here the MMD represents the distance between the mean embeddings of distributions in a reproducing kernel Hilbert space (RKHS), in our case, defined by a Gaussian kernel^12^ (Fig. 4a). If the extent of morphological change is reflected in the extracted numerical features, then one would expect a larger MMD score at concentrations with greater change in morphology, since the two classes should be more distinguishable. Figure 4b shows the change in the MMD against drug concentration for two experimental trials (see Methods), in which the pillars for both experimental results are similar. The trend of the MMD shown in Fig. 4b is also consistent with the trend of classification accuracy in Fig. 3b, demonstrating that the measured dose dependence in the SVM results is supported by the MMD score of the feature space. Note that in contrast to the histograms shown in Fig. 3a, in which the separation of two classes is made possible by supervised learning with respect to class labels, the MMD (the distance between the distributions of two classes) is computed in a closed form and therefore does not require supervised learning. As a larger MMD indicates a larger morphological change present in the distribution, it is more likely that the SVM model at the drug concentration giving the largest MMD assigns large weights to features that reflect this drug-induced morphological change. Accordingly, we also quantitatively analyzed the MMD for each feature at the drug concentrations in which the overall MMD between the two class distributions is largest (1 µM of the first experiment and 100 nM of the second experiment as shown in Fig. 4b). We computed the MMD of each feature to examine the variation between the two experiments. Figure 4c shows that the features giving larger MMD scores are highly correlated between the two experiments, indicating that the significant features are consistent in both experiments. It is also observed in Fig. 4c that features with large MMD score represent various types of information of cell images, such as geometry, granularity, intensity, and texture, indicating that the multivariate data provided by single-cell images is effective for identifying the cellular response to the drug.

We further investigated how the number of features contributes to the classification accuracy. We iteratively performed SVM classification between negative control and the data at 1 µM in the first experiment by removing the features with lower MMD (Fig. 4d). Consequently, we found that an accuracy of 90% (dotted line in Fig. 4d) was maintained when more than 400 features were removed, suggesting that with approximately 100 features, the performance of our label-free method is comparable to that obtained with fluorescence imaging techniques^4^. This property is noteworthy because fluorescence imaging requires fluorescent labeling with several inherent drawbacks as mentioned above. In other words, the high specificity of fluorescence imaging can also be provided by the combination of high-throughput bright-field imaging and machine learning, which is highly beneficial for pharmaceutical industry in which the cost of drug discovery is one of the major limiting factors.

### Dose-dependent classification accuracy with a single model

**Figure 5:**
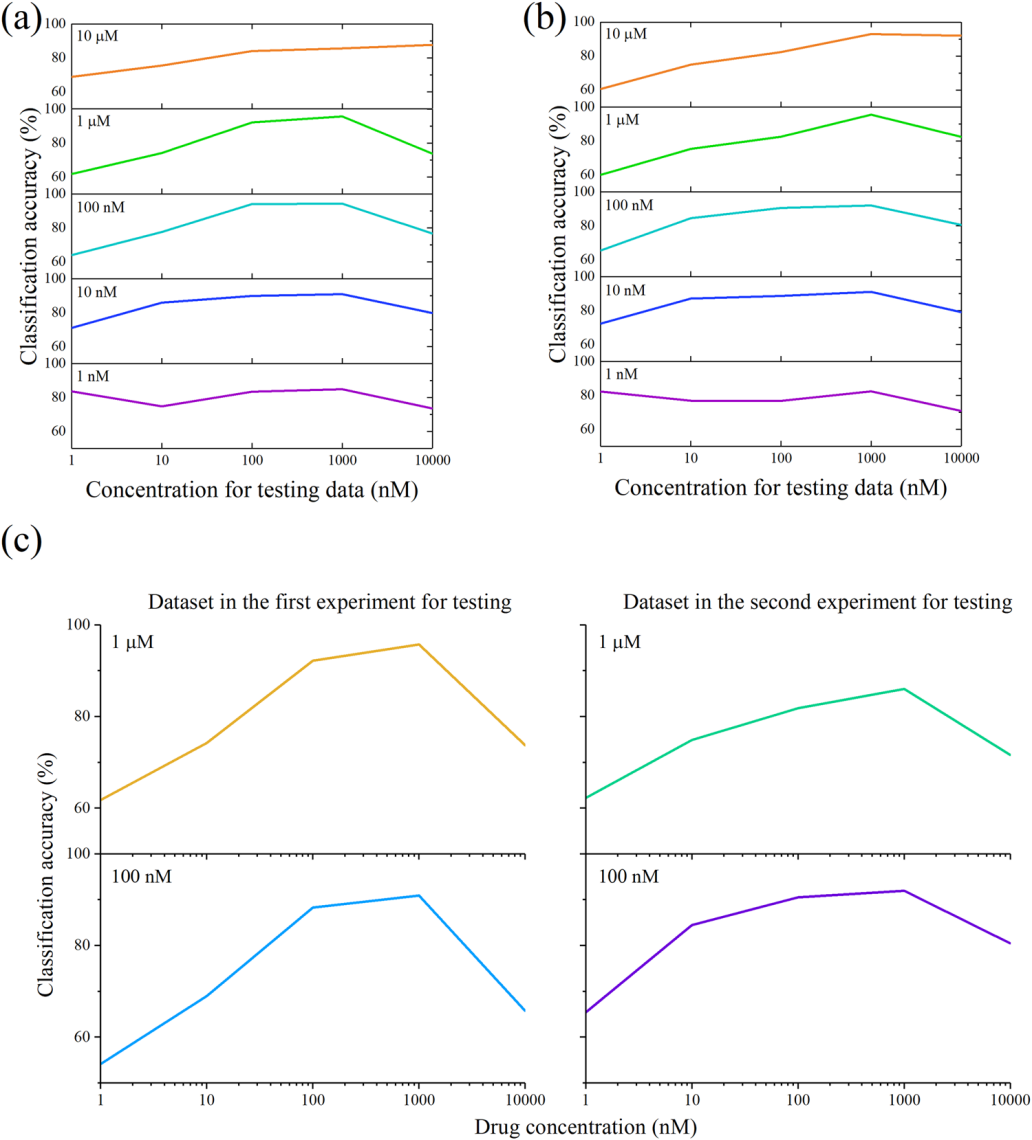
Classification accuracy using single SVM models. Classification accuracy produced by the SVM models in the first experiment (**a**) and the second experiment (**b**). Each row demonstrates the classification accuracy at each drug concentration produced by a single SVM model. (**c**) Evaluation of single SVM models across different experiments. Each row demonstrates the classification accuracy at each drug concentration produced by a single SVM model trained with the data from 1 µM in the first experiment (upper) and 100 nM in the second experiment (lower). Each column demonstrates the testing data from different trial of experiments.

We tested whether a single SVM model with a linear kernel can represent the dose dependence through the classification accuracy at all concentrations. We applied the SVM model trained at one specific concentration to all the other concentrations, and show the result in Fig. 5a and Fig. 5b. The result shows that the SVM models of those concentrations with larger MMD between two class distributions (such as 100 nM and 1 µM) can better preserve the tendency in classification accuracy as shown in Fig. 3b, whereas the SVM models of those concentrations with lower MMD (such as 1 nM) fail to demonstrate such trend. Therefore, it is reasonable to conclude that our approach can provide a single classification model exhibiting the dose dependence of drug-induced morphological change. In addition, we further tested whether the single SVM models can be applied to different experimental trials. Specifically, we applied the model trained at the concentration with the largest MMD in the first experiment to the dataset of the second experiment, and vice versa (Fig. 5c). For the sake of comparison, the classifications in which both training and testing data are from the same trial of experiment are also included in the Fig. 5c. The result shows a consistent tendency in classification accuracy regardless of whether the training and testing data are from different trials of experiment, suggesting that the SVM models trained at the concentration giving the largest MMD can demonstrate the dose dependence across multiple experiments. This is a significant property in comparison with most of the previous work where a new training is required for a new dataset of images^13^.

## Discussion

In this paper, we proposed and experimentally demonstrated a method to detect cellular drug responses via drug-induced morphological change which are inarguably too subtle for the human eye to identify, but are identifiable with the combination of numerous bright-field cell images and machine learning. By leveraging machine learning techniques, we captured these subtle changes and successfully distinguished drug-treated and -untreated cells only by the use of bright-field images at a high classification accuracy of 92% without the need for any labeling techniques. Furthermore, we also verified that our approach is capable of robustly capturing invariant and distinctive features in drug-induced morphological change, such that a one-time trained classifier model can be used for different datasets. This is a significant property for screening applications.

In this study, our optofluidic time-stretch microscope achieved a frame rate up to 250,000 frames/s, corresponding to an unprecedented throughput of 250,000 cells/s, which is 50 times higher than that of commercial imaging flow cytometers^14^. Despite its high throughput, the spatial resolution of our microscope remained at the diffraction limit of 780 nm^15^, which is comparable to that of conventional optical microscopes. To the best of our knowledge, this is the first experimental demonstration of an imaging flow cytometer capable of taking diffraction-limited images at a theoretical throughput of more than 100,000 cells/s, which is challenging even for conventional non-imaging flow cytometry^16–18^. The combination of such high-throughput and high-resolution capabilities allows us to use only bright-field images and simple machine learning algorithms to identify the miniscule drug-induced morphological change of cells with high accuracy.

While our method was demonstrated on MCF-7 cells as a proof-of-principle application, it is also applicable to other types of cells and drugs in various settings. Although MCF-7 cells are adhesive cells whose morphological change is typically observed when they are adhered on a surface^19,20^, we chose to observe their morphological change in suspension despite the risk of losing their morphological change during the process of trypsinization, because higher throughput can be achieved in the flow-cytometric manner. Nonetheless, we demonstrated that morphological change can also be investigated for the adhesive cells in a suspended condition with our approach, suggesting that it can be applied in distinguishing dissociated tissue samples via their morphological variations. As for the choice of the classifier, the SVM has been widely used in the field of bioinformatics for various applications including promoter determination^21^, protein remote homology detection^22^, and DNA bind protein prediction^23^. We chose a linear SVM classifier in this study for the proof-of-concept demonstration because it is a simple machine-learning algorithm and is unlikely to overfit. The simplicity of using the SVM allows one to easily perform classification without the support of a web server compared to other bioinformatic methods^24^. More advanced machine-learning algorithms can also be applied to achieve higher accuracy; for instance, ensemble methods^10^ or deep architectures^25,26^ can be used for classification. In addition, instead of hand-coding features, convolutional networks can be used to extract features that are problem-specific and therefore further improve classification accuracy.

## Materials and Methods

### Cell culturing and treatment

As a proof-of-principle demonstration of our method, we used a human breast adenocarcinoma cell line, MCF-7 (DS Pharma Biomedical) as model cells. The cells were maintained in Dulbecco’s Modified Eagle Medium (DMEM) (Wako Chemicals) supplemented with 10% fetal bovine serum (MP Biomedicals) and 1% penicillin streptomycin (Wako Chemicals), at 37 °C and 5% CO_2_. Paclitaxel, an FDA-approved anti-cancer drug, in a powder form (Cayman Chemical) was dissolved in dimethyl sulfoxide (DMSO, Wako Chemicals) to a stock concentration of 1 mM. One day after seeding, the cells were incubated with 10-fold serial dilutions (ranging from 1 nM to 10 µM) of paclitaxel, harvested at two intervals (12, 24 hours), and suspended in the culture medium by trypsinization for imaging with the time-stretch optofluidic microscope. A low cell concentration of approximately 10^5^–10^6^ cells/mL was used for the sample to ensure reliable single-cell image acquisition in each image frame.

### Optofluidic time-stretch microscope

The optofluidic time-stretch microscope is schematically shown in the left inset of Fig. 1 (analogous to the setups previously reported by us and others^25,27–33^). We used a Ti:Sapphire femtosecond pulse laser with a centre wavelength, bandwidth, and pulse repetition rate of 780 nm, 40 nm, and 75 MHz, respectively, as the optical source. Each laser pulse emitted from the laser was first temporally dispersed in a single-mode fibre spool with a total dispersion of -240 ps/nm, followed by spatial dispersion via a diffraction grating with a groove density of 1200 lines/mm. Next, the dispersed laser pulses were focused to the cells flowing in a microfluidic device via an objective lens with a numerical aperture (NA) of 0.6. The laser pulses bearing 1D cellular information were collected by another objective lens with the identical NA, spatially recombined by another diffraction grating identical to the first one, and detected by a high-speed photodetector (New Focus 1580-B with a bandwidth of 12 GHz). The photodetector signal was digitized by a high-speed oscilloscope (Tektronix DPO71604B with a bandwidth of 16 GHz and a sampling rate of 50 GS/s) to obtain 1D transmission images contained in the pulses. At last, the 2D images of the cells were obtained by digitally stacking the 1D images. To align the flowing cells in both the lateral and axial directions and avoid out-of-focus blurring, we employed hydrodynamic focusing in the microfluidic device to order and focus the cells during the measurement^34,35^. The dimensions of the microchannel were 100 μm in width and 44 μm in height. The total flow rate including the sheath flow and sample flow is 2.75 mL/min, resulting in a flow speed of approximately 10 m/s. Although the maximum possible throughput of the optofluidic time-stretch microscope is 250,000 cells/s (meaning that it acquires one cell image for every frame, given by the frame rate of 250,000 frames/s), the practical throughput lies between 10,000 and 100,000 cells/s, taking into account the cell concentration and the stability and durability of the microfluidic device in the microscope.

### Image processing and machine learning

Single-cell images were reconstructed and segmented on MATLAB (R2017a). A total number of 548 features, which consist of 43 geometry features (No. 1-43), 10 granularity features (No. 44-53), 43 intensity features (No. 54-96), and 452 texture features (No. 97-548), for each single-cell image were extracted with CellProfiler (ver. 2.2.0)^36,37^ and exported to our support vector machine (SVM) for cell classification on MATLAB. All classifications were performed by only using the unmodified functions provided in the Statistics and Machine Learning Toolbox on MATLAB. Approximately 10,000 single-cell images of MCF-7 cells were acquired for each concentration of drug treatment (including untreated negative control) during a single experiment. We repeated the experiment twice for two treatment durations of time (12, 24 hours), resulting in four groups of datasets including two groups of negative controls and two groups of treated cells in each treatment duration of time, which were used to implement four trials of SVM classification between negative control and drug-treated cells for each concentration. The total number of images acquired for this study is 240,000. All SVM classifications were performed with a linear kernel. The classification accuracies were estimated using 10-fold cross-validation.

### Maximum mean discrepancy (MMD) and feature selection

Maximum mean discrepancy (MMD) is used to measure the distance between two high-dimensional distributions^12^. Let *H* be the unit ball in a reproducing kernel Hilbert space (RKHS) and *k* be the corresponding characteristic kernel, the unbiased empirical estimate of squared MMD between distribution *X* of size *m* and distribution *Y* of size *n* is given by

2 $${{\rm{MMD}}}^{2}[ {\mathcal H} ,X,Y]=\frac{1}{m(m-1)}\sum _{i=1}^{m}\sum _{j\ne i}^{m}k({x}_{i},{x}_{j})+\frac{1}{n(n-1)}\sum _{i=1}^{n}\sum _{j\ne i}^{n}k({y}_{i},{y}_{j})-\frac{2}{mn}\sum _{i=1}^{m}\sum _{j\ne i}^{n}k({x}_{i},{y}_{j}).$$

Given that *X* and *Y* correspond to two classes we try to distinguish, a large MMD score indicates a large separation between the two distributions, and vice versa. In this work, *k* is selected to be the Gaussian kernel, which is known to be characteristic on real-valued distributions.

## Electronic supplementary material

Dataset 1
